# High-sensitivity polarization-independent terahertz Taichi-like micro-ring sensors based on toroidal dipole resonance for concentration detection of Aβ protein

**DOI:** 10.1515/nanoph-2023-0010

**Published:** 2023-02-24

**Authors:** Wencan Liu, Xinwei Zhou, Shucai Zou, Zhengguang Hu, Yun Shen, Mengqiang Cai, Dongdong Lin, Jia Zhou, Xiaohua Deng, Tianjing Guo, Jiangtao Lei

**Affiliations:** Department of Physics, School of Physics and Materials Science, Nanchang University, Xuefu Avenue 999, Nanchang City 330031, China; Institute of Space Science and Technology, Nanchang University, Xuefu Avenue 999, Nanchang City 330031, China; Department of Physics and Qian Xuesen Collaborative Research Center of Astrochemistry and Space Life Sciences, Ningbo University, Ningbo, Zhejiang 315211, China

**Keywords:** Aβ protein, metamaterial sensor, Taichi-like micro-ring, terahertz, toroidal dipole resonance

## Abstract

Terahertz (THz) metamaterial sensor is a newly-developing interdisciplinary technology, which combines the essential characteristics of THz spectroscopy and metamaterials, to obtain better sensitivity for trace detection of the different target analytes. Toroidal dipole resonances show great sensing potential due to their suppression of the radiative loss channel. Here, we found a high-quality planar toroidal dipole resonance in the breaking Chinese Taichi-like ring and then designed a novel polarization-independent terahertz toroidal sensor by combining four Taichi-like rings into a cycle unit. The sensor shows high-sensitivity sensing characteristics for the ultrathin analyte and refractive index. The optimized sensitivity of pure analytes under 4 μm coating thickness can numerically reach 258 GHz/RIU in the corresponding ∼1.345 THz frequency domain, which is much higher than that of previously reported sensors. We further fabricated experimentally the sensor and demonstrated its fascinating polarization-independent characteristics. Finally, it was successfully applied to the low-concentration detection (ranging from 0.0001 mg/mL to 10 mg/mL) of Aβ protein associated with Alzheimer’s disease. Our high-sensitivity polarization-independent THz toroidal dipole sensor would give access to rich applications in label-free biosensing.

## 1 Introduction

Terahertz (THz) spectroscopy has received growing attention in biomedical applications owing to its prominent characteristics of non-invasive, non-ionizing, low scattering, high penetrability, and fingerprint spectrum [1–6]. It can generally be applied to probe and characterize various biomolecules including nucleic acid [7, 8], protein [9], phospholipid [10], cell [11], and tissue [12]. However, the low sensitivity of free-space THz detector limits the trace detection of many biomolecules. Fortunately, THz metamaterials present tremendous opportunities to effectively extend the utilization of THz technology in the biosensing field by boosting light–matter interactions [13, 14].

THz metamaterials are periodic artificial electromagnetic media with extraordinary physical properties that cannot be found in nature, involving anomalous refraction [15, 16], negative permeability [17], super-absorption [18], cloakings [19, 20], etc… Besides those inspiring advances, the appearance of a spoof surface plasmon with localized electric field enhancement near the metal, as well as large values of quality factor (Q factor) [21], makes these metamaterials highly sensitive to minor environmental changes [13]. Researchers have shown that these properties generally are not derived from the physical properties of materials themselves but mostly depend on artificially constructed structures [22]. Therefore, a variety of THz metamaterial sensors based on different structures and corresponding physical rules have been proposed [23, 24]. For example, Wu et al. fabricated U-shaped golden split ring sensors with an inductor-capacitor (LC) and plasmonic dipole resonance to detect streptavidin–agarose [25]. Qin et al. designed circular apertures for the detection of tetracycline hydrochloride based on the extraordinary optical transmission (EOT) effect [26]. Zhang et al. fabricated an electromagnetic-induced-transparency (EIT) biosensor consisting of cut wires and split ring resonators to distinguish mutant and wild-type glioma cells [27]. Zhou et al. designed a label-free Fano-based THz microfluidic sensor for DNA molecule detection [28]. As sign-enhanced carriers, THz metamaterials are indeed very suitable to detect biomolecules and biological samples.

Among these THz metamaterials, toroidal dipole metamaterials have received growing attention and are considered as a novel approach to regulating intrinsic radiative losses in recent years [29]. Different from the traditional electric and magnetic dipoles, toroidal dipoles are characterized by the creation of a closed-loop configuration of the magnetic fields and currents rotating on the surface of a torus [30]. Researchers have shown that the Q factor and the figure of merit of the toroidal dipolar mode are significantly higher than traditional Fano resonance [31]. Meantime, THz toroidal metamaterial presents unique tunable properties, such as dynamically switching capacity to the fundamental electric dipole or magnetic dipole [30, 32]. Considering the unique advantages, THz toroidal metamaterials have been explored for the ultra-trace detection of biomolecules. Ahmadivand et al. achieved rapid detection of infectious envelope proteins by magnetoplasmonic toroidal metasensors [33]. Wang et al. reported ultrasensitive THz sensing with high-Q toroidal dipole resonance governed by bound states in the continuum (BIC) in an all-dielectric metasurface [34].

Alzheimer’s (AD) is the most prevalent neurodegenerative disease and mainly characterized by the deposition of extracellular plaque composed of amyloid-β protein (Aβ) [35, 36]. The Aβ levels in the blood are efficacious in predicting the severity and progression at early or preclinical stages of AD [37]. Various fluorescent biosensors are developed to provide Aβ pathology information [38]. However, fluorescence labels such as Congo red and thioflavin-T analogs have been found to inhibit further fibrillization due to Aβ binding [39], thus affecting the accuracy of the detection. Recently, label-free THz near-field spectroscopy can be used to identify the fibrillization state of Aβ protein [40]. Our group also reported that THz metal-graphene hybrid metamaterial can monitor the aggregation of Aβ_16–22_ peptides by successfully linking the Fermi level change of graphene to the shifting Rabi splitting peaks [41]. It is of great significance to further develop new THz metamaterials to detect Aβ concentrations more accurately.

Considering the Q factor and the measurement stability are always affected by the electric field polarization of the incident THz wave [42], we introduced symmetry breaking into the centrosymmetric Chinese Taichi-like ring, with which a high-quality planar toroidal dipole response model can be excited. Thus, a novel polarization-independent THz toroidal sensor can be designed by combining four Taichi-like rings into one unit with a 90° rotation. The sensor shows high sensitivity to environmental perturbations, such as the ultrathin thickness and refractive index of the analyte layer. The optimized sensitivity of pure analytes under 4 μm coating thickness can numerically reach a high value of 258 GHz/RIU in the corresponding ∼1.345 THz frequency domain, showing a high sensitivity characteristic. Then, we fabricated experimentally the sensor and demonstrated its attractive polarization-independent characteristics. Finally, it was successfully applied to the low-concentration detection of Aβ protein ranging from 0.0001 mg/mL to 10 mg/mL. Our high-sensitivity polarization-independent THz toroidal dipole sensor would give access to rich applications in the diagnosis of related diseases.

## 2 Results and discussion

### 2.1 Structural design of THz Taichi-like ring metamaterial with toroidal dipole resonance

**Figure 1:**
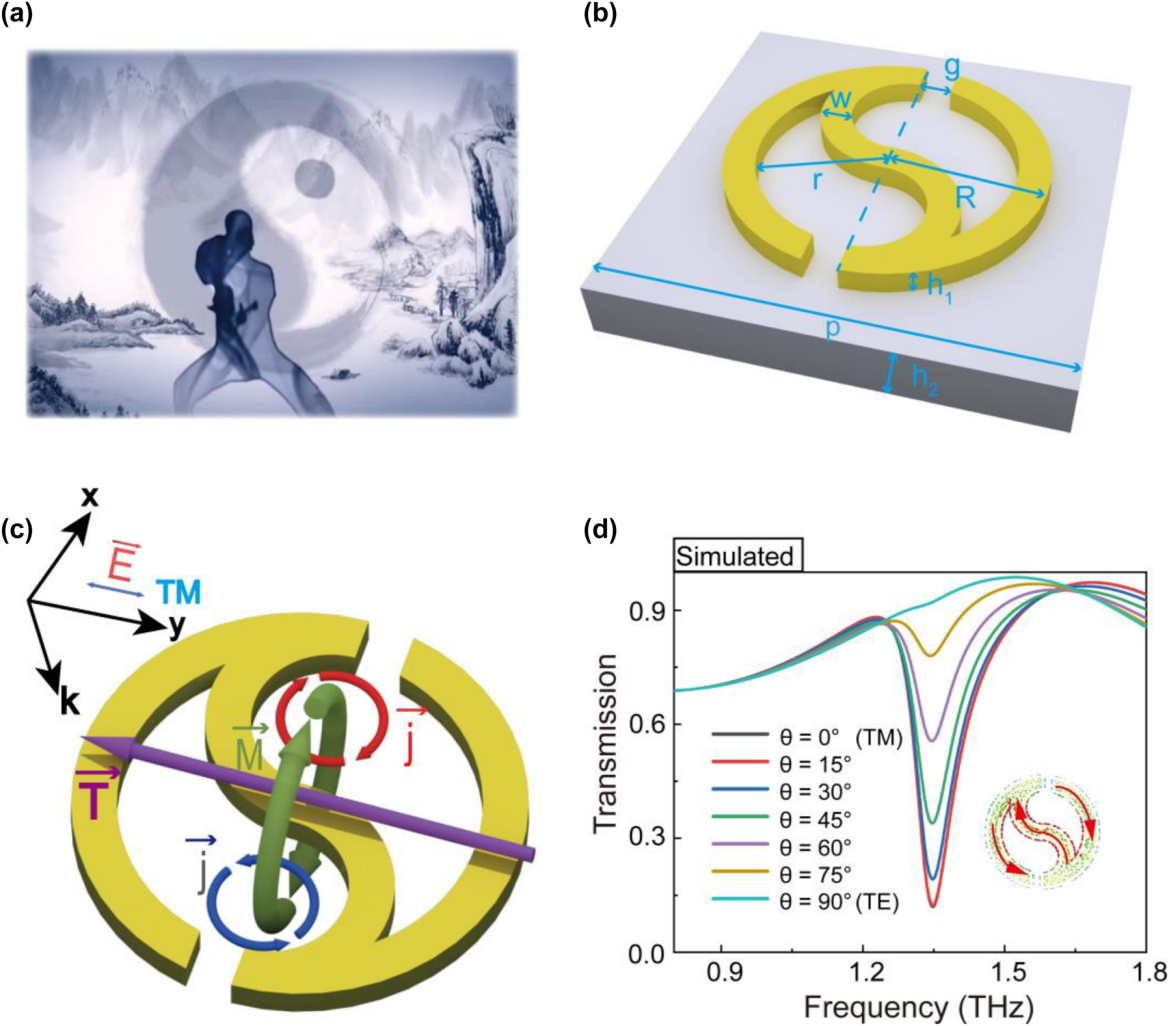
Structural design of a single Taichi-like ring for THz toroidal dipole metamaterial. (a) Taichi diagram in ancient and mysterious Chinese culture. (b) Schematic of the single Taichi-like ring (STR) metasurface. (c) Toroidal dipole resonance generated by the STR under TM polarization. (d) Transmission spectra of the STR metasurface under different polarized light incidents.

Many studies have shown that strong toroidal resonances can be aroused by extraordinary 2D metamaterials with perfect symmetric and breaking geometries [31, 33, 43]. Inspired by a large symmetric Taichi diagram from ancient and mysterious Chinese culture (shown in Figure 1a), we designed a novel high-Q planar toroidal dipole response metamaterial by introducing symmetry breaking into the Taichi ring. As shown in Figure 1b, the half of a single Taichi-like ring (STR) is wide in size at the head and gradually becomes small at the tail, and the designed unit cell of the resonator is a single ring with a split gap at the upper and lower heads. The structural parameters are as follows: the STR has an outer radius of *R* = 14 μm, an inner radius of *r* = 11 μm, a split gap of *g* = 3 μm, a line width of *w* = 3 μm (*w* = *R* − *r*) and a thick of *h* = 200 nm. The metamaterial resonator consists of copper (Cu) STR arrays with a period of *p* = 80 μm on a 50 μm thick polyimide (PI) substrate.

When the STR is normally illuminated by an incident THz beam with TM polarization (where the incident electric field is parallel to the *y*-axis), the surface currents (**j**) along the superatom are excited, and the current loops construct magnetic dipoles in opposite directions, and thus a head-to-tail magnetic field (**M**) is constructed and confined (seen in Figure 1c). According to the right-hand rule, such a loop magnetic field results in a toroidal dipole vector along the *y*-axis, shown as the purple arrow in Figure 1c. Detailed simulation results of the surface currents (**j**) and magnetic field (**M**) are presented in the inset of Figure 1d and Figure S1 in the Supplementary material, respectively. Figure 1d exhibits the transmission spectra of STR illuminated by incident THz beam with different polarized angles ranging from 0° to 90°. The transmission spectrum with TM polarization shows the largest resonance dip at 1.345 THz, where the spins made up of surface currents are in opposite directions (inset in Figure 1d), thus forming a toroidal dipole resonance. Though THzlight with different polarizations (except the TE model) can stimulate a resonance dip at 1.345 THz, the quality factor will reduce with the increased angle.

### 2.2 Polarization-independent characteristics of quadruple Taichi-like rings

**Figure 2:**
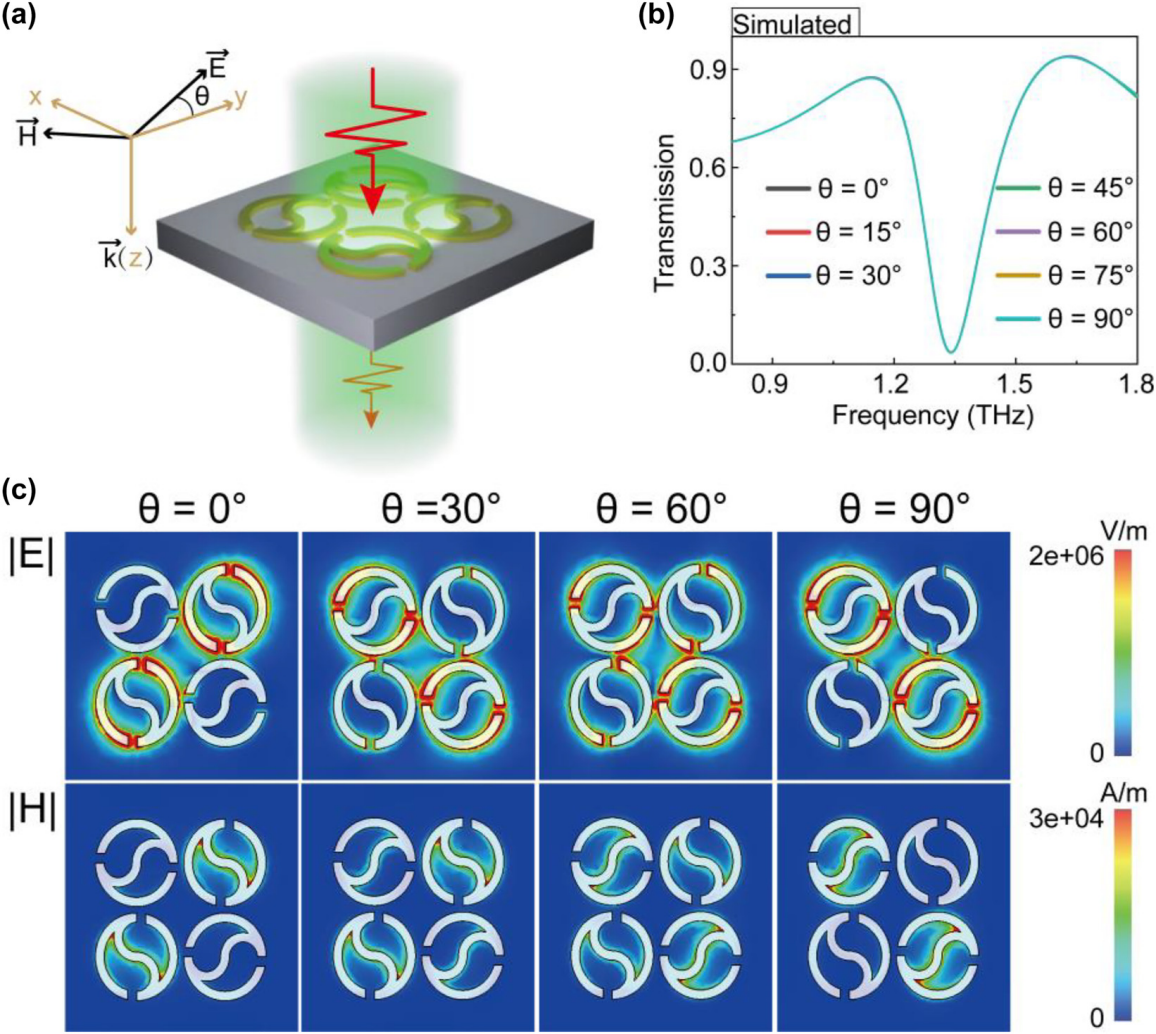
Quadruple Taichi-like rings (a) schematic of QTR metasurface with the incident polarization angle *θ*. (b) The transmission spectra of the QTR metasurface illuminated by incident light with multiple polarization angle *θ*. (c) Near electromagnetic field distribution of polarization excitation with various angles at toroidal resonance frequencies.

In order to eliminate the quality-factor degradation caused by the polarization angle of incident THz light, we further designed a novel polarization-independent THz toroidal sensor by combining four Taiji rings into a cycle unit with an unchanged period. As shown in Figure 2a, the two pairs of diagonal structures were perpendicular to each other and the unit cell thus has group C4 symmetry. For the quadruple Taichi ring (QTR) metasurface, the superatoms are symmetric along the *x*-axis and *y*-axis, which is expected to lead to the same frequency response under different polarization incidences. To verify this conclusion, we simulated the transmission spectra of the QTR metasurface illuminated by incident light with multiple polarization angles. It turns out that these transmission spectra overlap very well, as shown in Figure 2b, indicating that the toroidal resonance response of QTR metasurface is polarization-independent.

To further study the physical mechanism of polarization-independent characteristics, the electromagnetic field distributions at polarization angles of 0°, 30°, 60°, and 90° were monitored at the resonance frequency, as shown in Figure 2c. When the polarization angle is 0°, the incident light only excites the surface plasma resonance of the two Taichi-like rings on the diagonal in the supercell. Meanwhile, we noted that the areas with electric field enhancement and magnetic field enhancement are mainly located at the heads and the tail of Taichi-like ring, respectively. With the variation of polarization angle, the excitation intensity of the surface plasma resonance gradually transits to the other diagonal Taichi-like rings. These results indicate that our designed structure presents polarization-independent characteristics.

### 2.3 High-sensitivity sensing characteristics for ultrathin analyte

**Figure 3:**
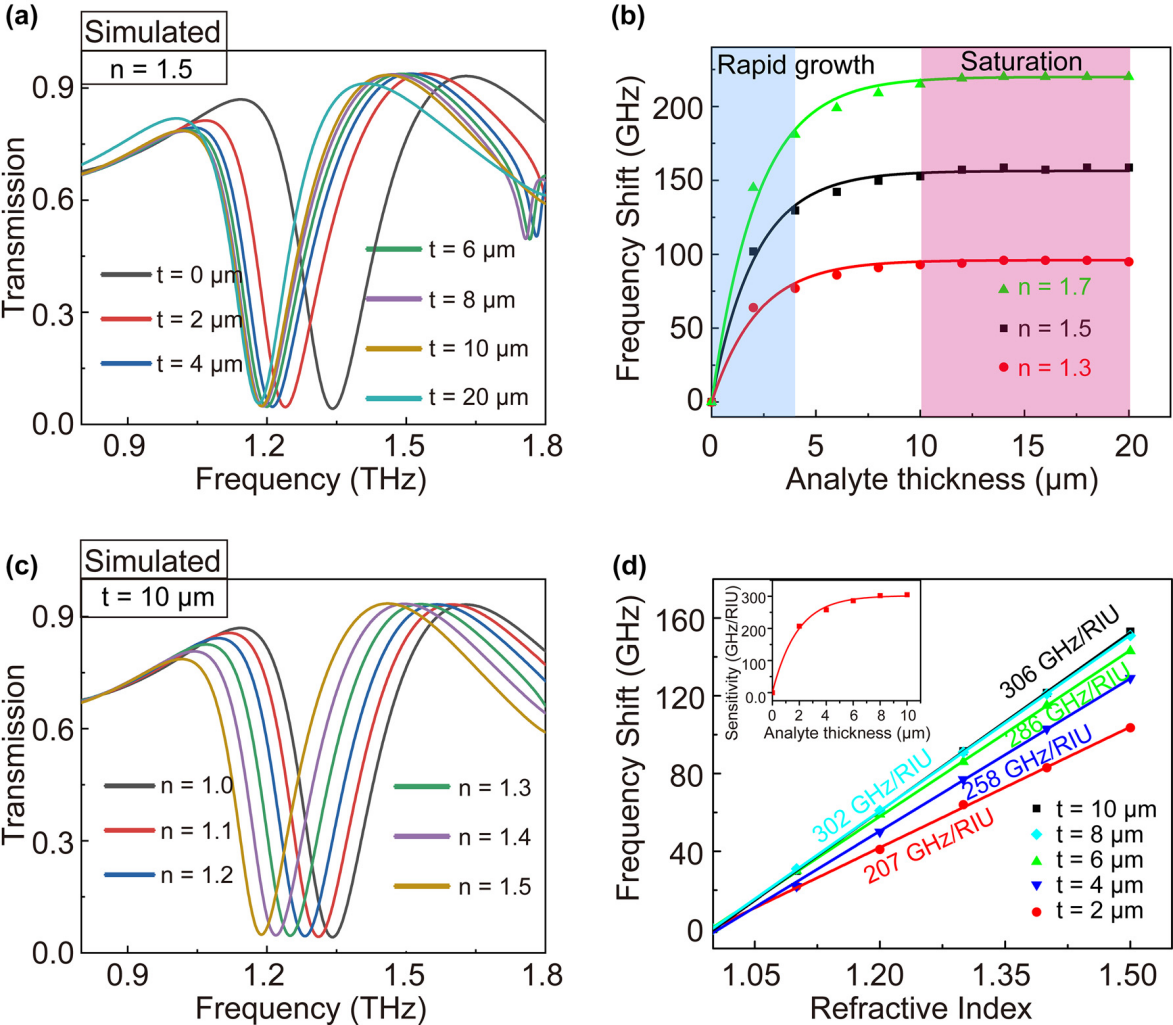
The analyses of sensing characteristics. The simulated transmission spectra of QTR metasurface with the variation of (a) thickness (*n* = 1.5) and (c) refractive of the analyte (*h* = 10 μm). (b) The frequency shift of QTR metasurface versus the thickness of analyte (*n* = 1.5). (d) The frequency shifts of QTR metasurface versus the refractive index with different thicknesses of the analyte. Linear fit has been performed to determine the sensitivity of the metasurface.

To investigate sensing characteristics for biosensing applications, we simulated the variation of transmission spectra with the refractive index *n* and thickness t of the covered analyte. Figure 3a shows the dependence of THz spectra on the analyte thickness when an analyte with a refractive index of 1.5 is deposited on the surface of the QTR metasurface. The transmission spectra present a gradual redshift from 1.345 to 1.187 THz, as the analyte thickness increases from 0 μm (without analyte) to 20 μm. The relative frequency shift Δ*f* is calculated as *f*(*t*)−*f*(*t*_0_), where *f*(*t*) is the resonance frequency with analyte thickness *t*, and *f*(*t*_0_) is the resonance frequency without the analyte. As shown in Figure 3b, frequency shift Δ*f* rapidly increases within the first 4 μm analyte thickness and then achieves slowly a saturation state until 10 μm, showing that Δ*f* as a function of analyte thickness satisfies saturation nonlinearity. This phenomenon meets the localization effect of the electromagnetic field of the local surface plasma resonance.

Subsequently, we numerically calculated transmission spectra as the refractive index of the analyte increased from 1.0 to 1.5, by remaining a saturated thickness of 10 μm. The results were shown in Figure 3c. It can be seen that the resonance dip shows a redshift from 1.345 to 1.192 THz with the refractive index varying from 1 to 1.5. As shown in Figure 3d, different from the transformation law of frequency shift Δ*f* versus the thickness, the fitting function Δ*f* versus refractive index satisfies an almost linear relation. The refractive index sensitivity *S* can be calculated as *S* = Δ*f*/Δ*n*. For the analyte with a saturated thickness of 10 μm, the largest theoretical sensitivity reaches up to 306 GHz/RIU (RIU, Refractive Index Unit). However, biomolecular analyte generally is extremely thin in biosensing applications. Therefore, we pay more attention to the sensitivity of the ultra-thin analyte. It is important to note that the sensitivity trends to nonlinear (exponential) saturation of growth with the increased thicknesses (inset of Figure 3d). The sensitivity *S* = 258 GHz/RIU (with *t* = 4 μm) of our QTR metasurface has reached 84% of its saturation, showing its great superiority in the detection of ultrathin analytes.

**Figure 4:**
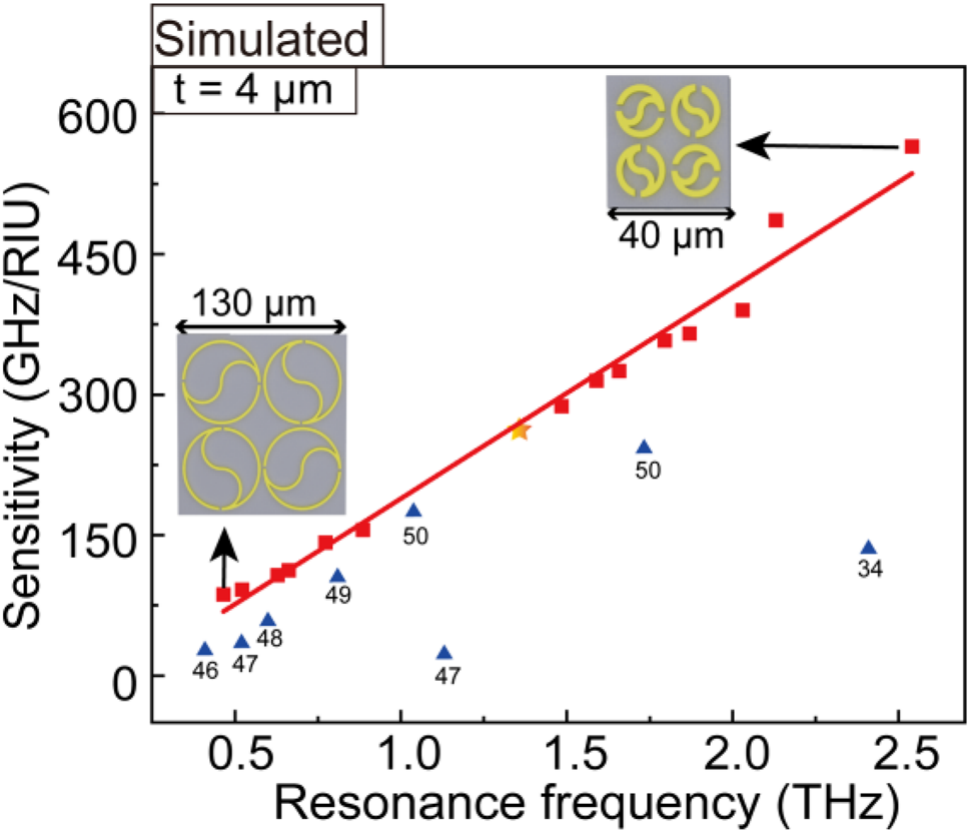
Refractive index sensitivity with varying resonant frequencies for 4 µm thick analyte. Our QTR metasurfaces are shown as red squares (The aforementioned structure is specifically marked as a yellow five-pointed star). Compared metasurface sensors are shown as blue triangles and the serial number of corresponding references is marked.

To further assess the level of refractive index sensitivity of our proposed metasurface for the ultrathin analyte, we compared the performance with those of reported state-of-the-art THz metasurface sensors. According to perturbation theory [44, 45], the frequency shift Δ*f* of the metasurface is not only related to the thickness and refractive index of the analytes but also the resonant frequency *f*_0_. The frequency shift Δ*f* is generally large with high resonance frequency. Hence, it is unreasonable or rigorous to show the superiority of our structure by merely comparing the value of refractive index sensitivity *S* = Δ*f*/Δ*n* but ignoring the thickness and the resonance frequency *f*_0_. In Figure 4, we changed the resonance frequency *f*_0_ from 0.465 and 2.540 THz by adjusting the geometrical parameters of the QTR metasurface (shown in the inset of Figure 4). When the thickness *t* = 4 μm, the refractive index sensitivities are approximately proportional to the resonant frequency *f*_0_ (ranging from 0.465 to 2.540 THz). Ultimately, a comparison of the performance of our proposed metasurface to those of reported state-of-the-art THz metasurface sensors [34, 46], [47], [48], [49], [50] is presented in Figure 4. Our structure has a high sensitivity for 4 μm thick analytes and shows its great superiority in sensing applications over the previous works. These results demonstrate that our QTR ring dipole resonant metasurface is extremely sensitive to the small change in thickness and refractive index of ultrathin analyte and thus enhances its application potential for the detection of biomolecules.

### 2.4 Experimental preparation and validation

**Figure 5:**
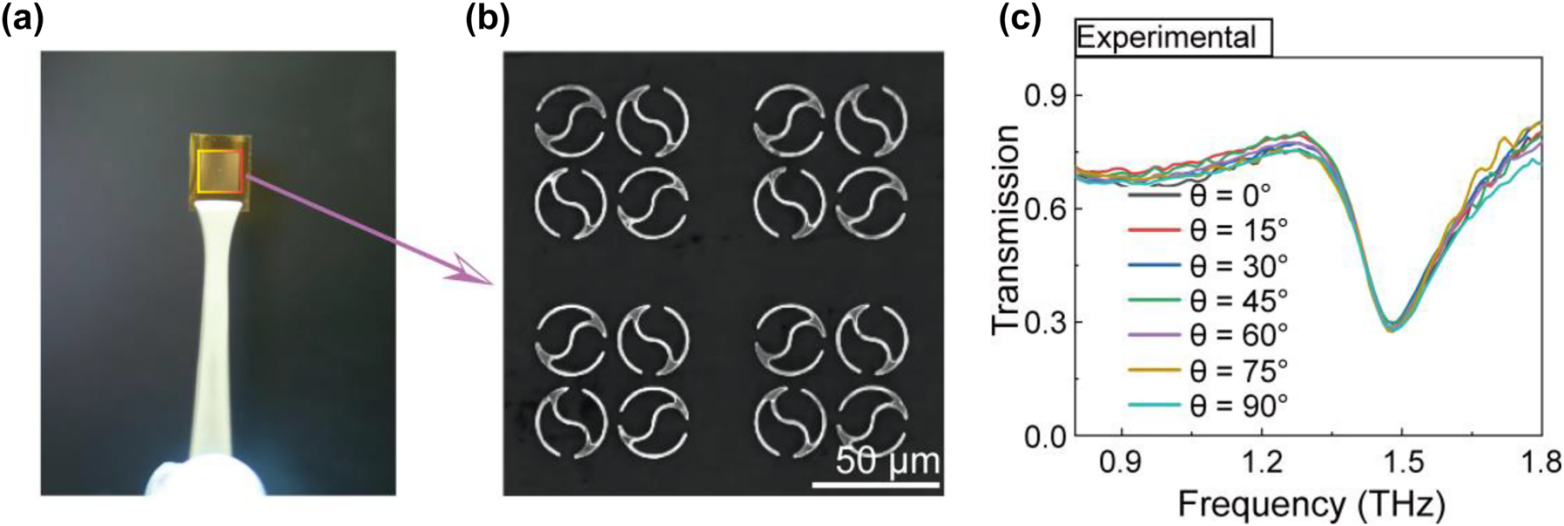
Polarization-independent behavior of QTR metasurface in the experiments (a) metasurface structure on PI substrate. (b) Microscopic image of QTR ring metasurface. (c) Experimental verification of resonant frequency response for different polarization angles.

To verify the proposed structure, a 200 nm thick 80 × 80 QTR array was fabricated by photolithography on a 50 μm thick polyimide film (Figure 5a). The detailed fabrication method is shown in Supplementary Figure S3. An optical microscopy image of the metasurface is shown in Figure 5b. We first investigated the independence of QTR’s toroidal resonance on the polarization angles of the THz beam by measuring the transmission spectra with a commercial THz time-domain spectroscopy (THz-TDs) system (BATOP-TDS1008). As seen in Figure 5c, the resonance frequency dip of the transmission spectrum under Ey-polarized (*θ* = 0°) THz waves emerged at 1.490 THz. As the variation of polarization angles of the THz beam from 0° to 90°, the transmission spectra and their resonance frequency dip overlap very well, consistent with the simulational results (Figure 2b). Compared to the numerically simulated spectra (Figure 2b), the experimental resonant intensity decrease and frequency blueshift with the value of 0.145 THz. The differences result from the slight absorption of polyimide film and the structural parameter deviation between the simulation and experiment. With the variation of the split gap, the tail, the outer radius and the inner radius, the resonance frequency shows a blue or red shift in the transmission spectrum (Figure S3). In addition, the roughness of the structure in the experiment would increase the Ohmic loss, which is reflected in the lower Q factor than the simulated transmission spectrum. Despite these biases, these experimental results still confirm that the toroidal resonance of our designed QTR is independent of the polarization of the incident light.

### 2.5 Concentration detection of Aβ protein

**Figure 6:**
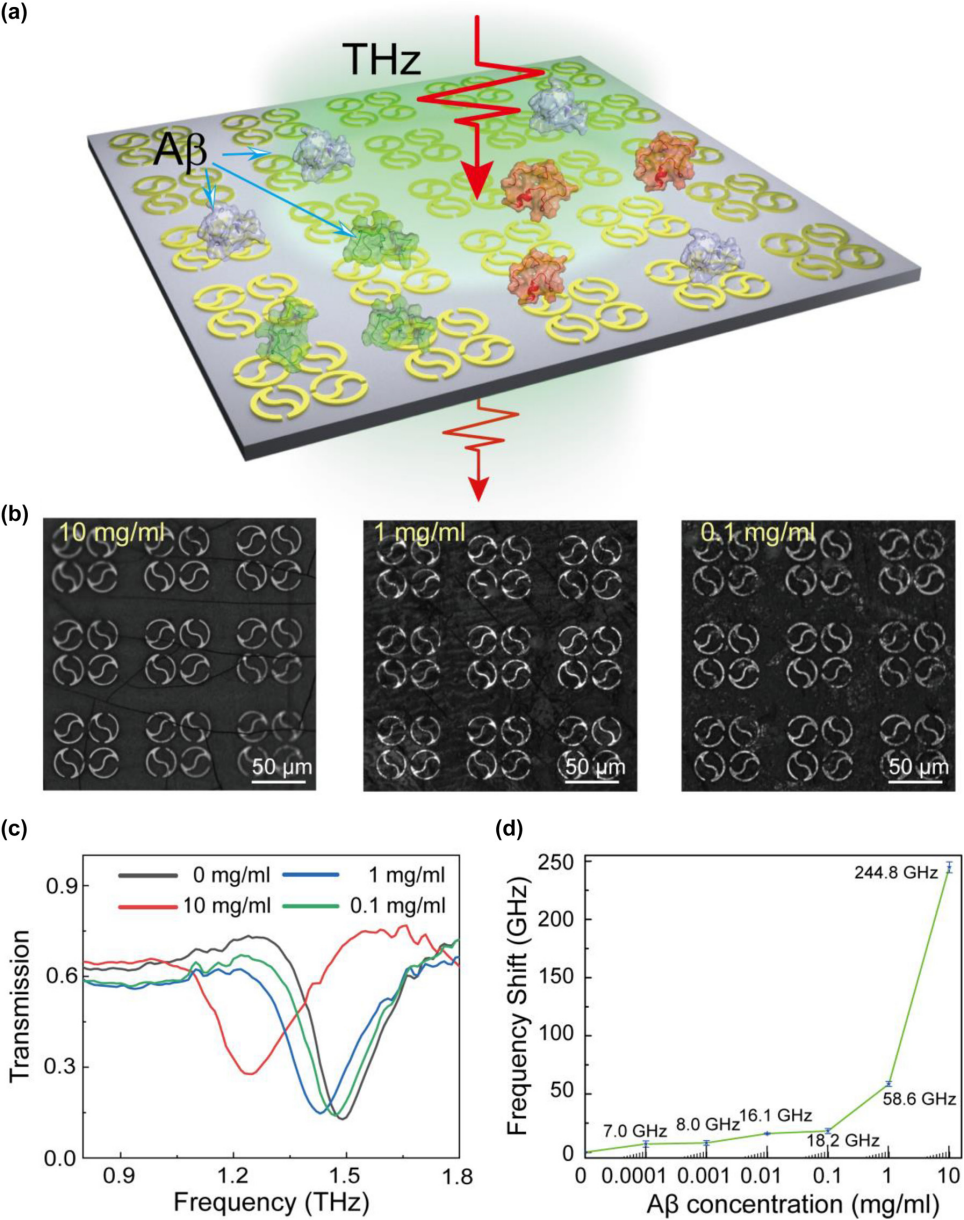
Concentration detection of Aβ protein. (a) Schematic illustration of QTR metasurface biosensor with Aβ protein. (b) Microscopic images of Aβ protein deposited on the metasurface with different concentrations. (c) The measured transmission spectra of Aβ protein deposited on the metasurface with different concentrations. (d) The frequency shift versus different concentrations of Aβ protein.

To investigate the performance of our QTR metasurface for biosensing applications, Aβ proteins with different concentrations were chosen as the analytes. Figure 6a shows a schematic illustration of Aβ protein deposited to the structure surface. Aβ solution samples with concentrations of 10 mg/mL, 1 mg/mL, 0.1 mg/mL, 0.01 mg/mL 0.001 mg/mL, and 0.0001 mg/mL were dropped onto the QTR surface, and then are quick-freezed at −80 °C after five-minute waiting and finally are freeze-dried at −20 °C in vacuum. Aβ aggregation involves the formation of different species of aggregates, including oligomers, protofilaments and mature fibrils [51]. The transition from monomers to initial oligomers, subsequent protofilaments, and eventual fibrils is considered to follow a nucleation–elongation process, consisting of three phases: lag phase, elongation phase, and steady phase [52, 53]. A large number of experiments show that the whole aggregation process generally needs to spend several hours [54–56]. In our experiment, we strictly control the incubation time in a matter of five minutes. As in any kinetical reaction, the Aβ fibrillization is strongly dependent on the temperature [57]. Therefore, quick-freezing (−80 °C) and vacuum freeze-drying (−20 °C) technique was used to block the Aβ aggregation and maintain its aqueous states as much as possible. As a result, our sensor primarily is used to detect Aβ monomers in the early stage of aggregation through these steps. The micrographs of protein analytes with the first three concentrations are shown in Figure 6b. The thin layers of Aβ protein with high concentrations of 10 mg/mL and 1 mg/mL and their drying cracks can be seen on the sensor surface. As the concentration decreases from 0.1 mg/mL to 0.0001 mg/mL, the reduction of sporadic protein accumulations could be observed (Figure 6b and Figure S4). Transmission spectra of QTR metasurface with Aβ protein were measured using THz-TDs system (Figure 6c). It can be seen that the resonance frequency of QTR metasurface without Aβ analyte coverage is 1.490 THz. As Aβ is covered by 10 mg/mL, 1 mg/mL, and 0.1 mg/mL, the resonance frequency blueshifts to 1.245, 1.431, and 1.482 THz, respectively. The reason for blueshift is that the variation of Aβ concentration covered on the surface of the structure shows a great effect on the equivalent refractive indices and thicknesses. The variation curve of resonance frequency shift versus Aβ concentration is shown in Figure 6d and all error bars also are shown in Supplementary Tables S1. Limited to the resolution ratio of our THz TDS system, the lowest detection limit of Aβ concentration is 0.0001 mg/mL with a 7 GHz frequency shift.

To assess the sensing level of our QTR metasurface biosensor for Aβ protein, we compared detection range with those of some representative biosensors. As seen in Table 1, the conventional detection sensors of Aβ include colorimetric [58, 59], electrochemistry [60, 61], fluorescent [62, 63] and immune [64, 65] sensor. The lower limit of detection of our QTR metasurface biosensor is comparable to that of colorimetric, electrochemistry and fluorescent biosensor but not as good as that of these immunosensor. The results indicate that our sensor indeed has great potential for detection of Aβ.

**Table 1:** Comparison of the representative sensors for detection of Aβ protein.

Method	Signal element	Detection range	References
Colorimetric	Au nanoparticles	10.5 nM–313.5 nM	[58]
	Au nanoparticles	35 nM–700 nM	[59]
Electrochemistry	Carbon nanotubes, Au nanoparticles and gelsolin-Au-Th bioconjugate	0.2 nM–40 nM	[60]
	Au nanoparticle on carbon electrode	0.5 μM–10 μM	[61]
Fluorescent	Resveratrol and graphene oxide	0–200 μM	[62]
	Polydopamine nanospheres	20 nM–10000 nM	[63]
Immunosensor with surface plasmon resonance	Au films and antibody	0.02 nM–5 nM	[64]
	Au substrate, Au nanoparticle, and antibody	1 fg/mL–10^9^ fg/mL	[65]
THz metamaterial	Cu QTR arrays	0.0001 mg/mL–10 mg/mL (23 nM–2.3 × 10^3^ μM)	This work

## 3 Conclusions

In conclusion, we found that a high-quality planar toroidal dipole response model can be excited from a symmetry-breaking centrosymmetric Taichi-like ring. To eliminate the quality-factor degradation caused by the polarization angle of incident THz light, we further designed a novel polarization-independent THz toroidal sensor by combining four Taichi-like rings into a cycle unit with a 90° rotation. The QTR sensor shows high-sensitivity sensing characteristics for the ultrathin analyte and different refractive indices. The optimized sensitivity of pure analytes under 4 μm coating thickness can numerically reach a high value of 258 GHz/RIU in the corresponding ∼1.345 THz frequency domain. Compared to reported THz metasurface sensors under the same condition, our structure has a higher sensitivity for 4 μm thick analytes. Then, we fabricated experimentally the sensor to verify our simulation results and demonstrated its attractive polarization-independent characteristics. Finally, it was successfully applied to the low-concentration detection of Aβ protein ranging from 0.0001 mg/mL to 10 mg/mL. These results indicate that the presented QTR metasurface has great sensing characteristics and promising applications for label-free biosensing.

Different biological samples have different physical properties such as refractive index. It is achievable to distinguish pure biological samples by using our all-metal THz metasurface sensors. For example, Chiben Zhang et al. has reported terahertz toroidal metasurface biosensor for the sensitive distinction of various lung cancer cells [66]. Jin Zhang et al. have shown a metamaterial-based biosensor for the recognition of molecule types of glioma cells [27]. However, it is limited to realizing biological sample detection in a hybrid system via using all-metal sensors due to the lack of specific binding surfaces. The functionalization of THz metasurface sensors by modifying and fixing special functional groups will be a very promising route for practical use.

## Supplementary Material

Supplementary Material Details

Supplementary Material Details
