# Supplementary material for: High-sensitivity polarization-independent terahertz Taichi-like micro-ring sensors based on toroidal dipole resonance for concentration detection of Aβ protein
Source: Nanophotonics. 2023 Feb 24;12(6):1177–87. doi: 10.1515/nanoph-2023-0010 (PMC11501499; doi:10.1515/nanoph-2023-0010)
Supplement: Supplementary file 1 — Supplementary Material Details [file j_nanoph-2023-0010_suppl_001.docx]

**High-sensitivity polarization-independent terahertz** **Taichi-like micro-ring sensors based on toroidal dipole resonance for concentration detection of Aβ protein**

Wencan Liu^a,b^, Xinwei Zhou^a,b^, Shucai Zou^a^, Zhengguang Hu^a^, Yun Shen^a^, Mengqiang Cai^b^, Dongdong Lin^c^, Jia Zhou^b^, Xiaohua Deng^a,b^, Tianjin Guo ^b^** and Jiangtao Lei^a,b^*

^a^Department of Physics, School of Physics and Materials Science, Nanchang University, Xuefu Avenue 999, Nanchang City 330031, China

^b^Institute of Space Science and Technology, Nanchang University, Xuefu Avenue 999, Nanchang City 330031, China

^c^Department of Physics and Qian Xuesen Collaborative Research Center of Astrochemistry and Space Life Sciences, Ningbo University, Ningbo, Zhejiang 315211, China

The authors declare no competing financial interest.

*Corresponding author: Jiangtao Lei, E-mail: jiangtaolei@ncu.edu.cn;

**Corresponding author: Tianjin Guo, E-mail: tianjin@ncu.edu.cn.

**This material contains three supplemental figures.**


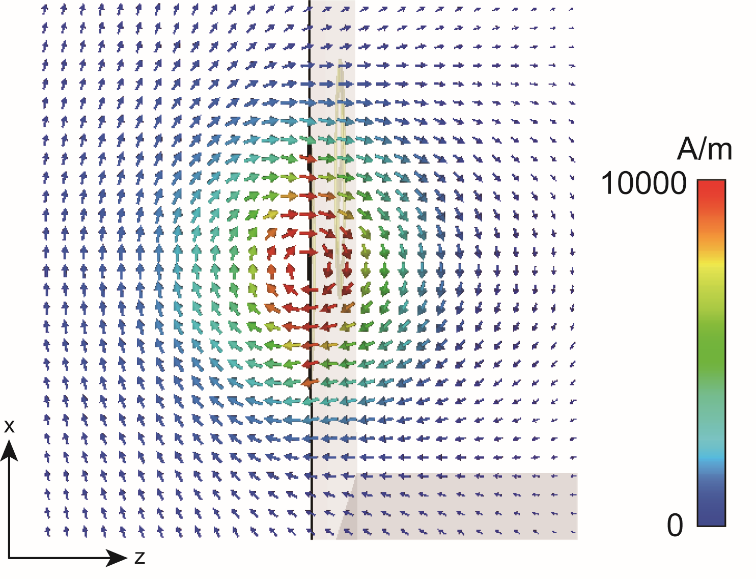


**Figure S1.** The magnetic field in the XZ plane。

**Fabrication of quadruple** **taichi ring (QTR) metasurface**

Figure S2 shows the fabrication of a quadruple taichi ring (QTR) metasurface. First, a PI film was fixed on a 500 µm thickness silicon slab by Ploydimethysiloxane (PDMS). Second, a layer of 200 nm thick Cu was deposited on the PI film by conventional electron-beam evaporation. Then, positive photoresist (SUN-115P) was coated on the PI film via the spin-coating method with a spin speed of 3000 rpm for 30 s and subsequently was baked at 100 ^◦^C for the 60 s. Next, direct laser writing lithography was used to expose the photoresist. The structure was baked at 100 ^◦^C for 90 s and placed into SUN-238D developer for 5 s. The exposed Cu was etched by reactive ion etching. Finally, photoresist residue was removed by using the acetone solution, and a flexible PI with quadruple taichi ring (QTR) metasurface was peeled off from the silicon slab.


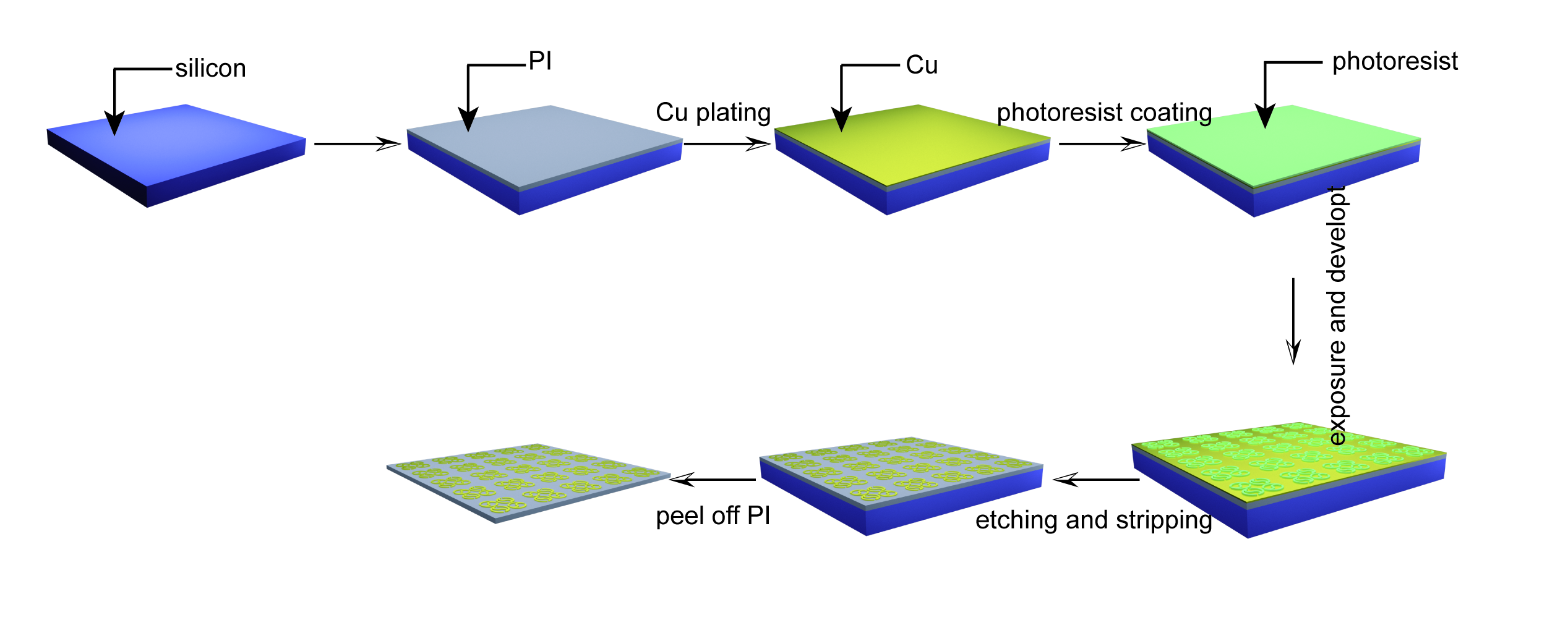


**Figure S2.** Fabrication of quadruple taichi ring (QTR) metasurface.


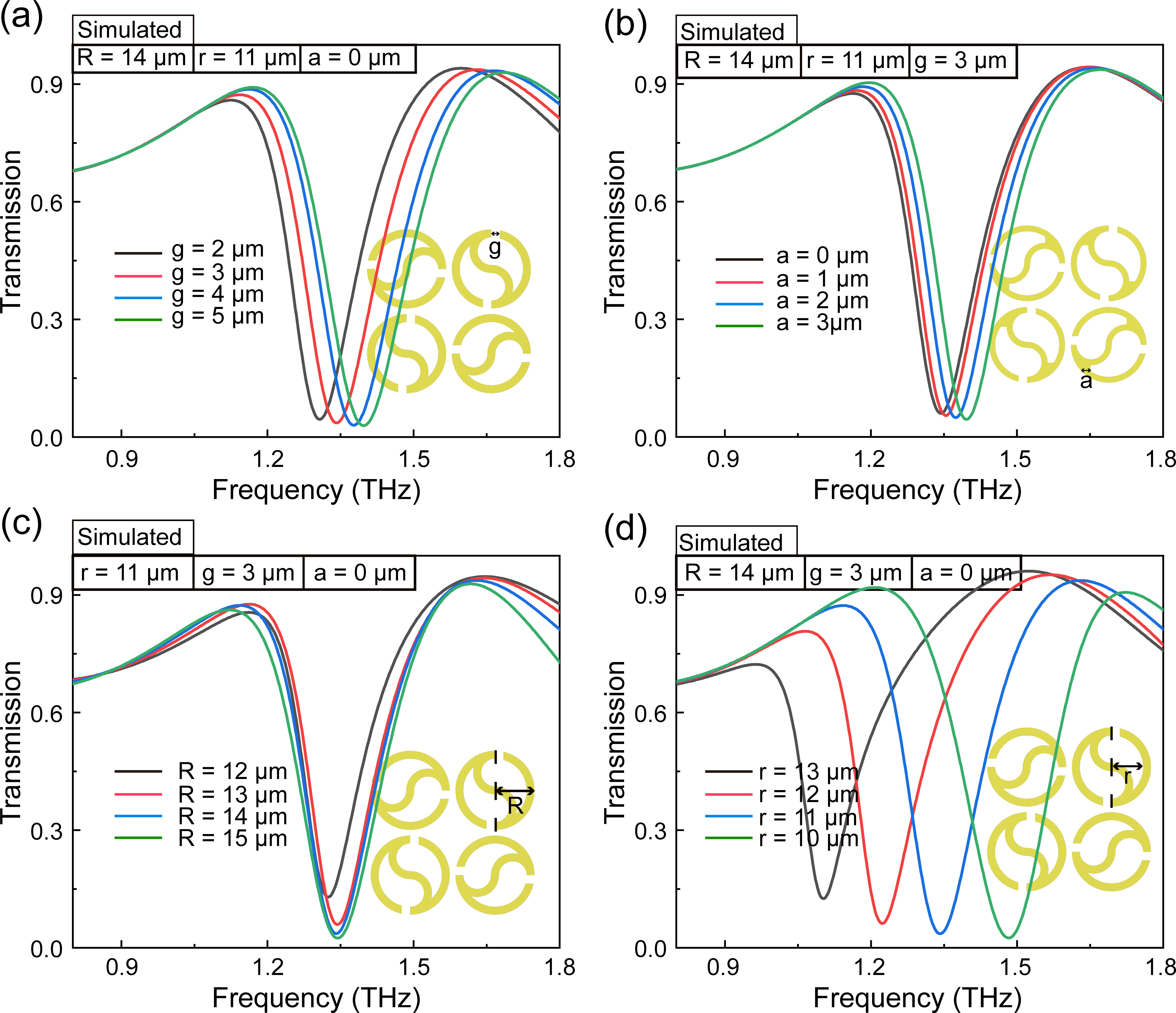


**Figure S3.** The simulated transmission spectra of QTR metasurface with the variation of (a) split gap. (b) the sharpness of the tail. (c) outer radius. (d) inner radius, respectively.

**Preparation of Aβ samples**

Highly purified (purity > 95%) full-length Aβ (residues 1~40) was obtained from Hangzhou Chinese Peptide Company. The Aβ solutions with 10 mg/ml concentration were prepared in pure water solution (pH 7.1) at room temperature. The solution was then gradually diluted to 1 mg/ml, 0.1 mg/ml, 0.01 mg/ml, and 0.001 mg/ml. The Aβ solutions are added to the surface of the structure in metamaterials and then are quick-freezed at -80 °C after five-minute waiting. They finally are freeze-dried at -20 °C in vacuum.
